# Diagnostic Accuracy of Five Molecular Assays for the Detection of Dengue Virus

**DOI:** 10.3390/medicina60091557

**Published:** 2024-09-23

**Authors:** Marianna Scarpaleggia, Giada Garzillo, Miriana Lucente, Chiara Fraccalvieri, Nadia Randazzo, Elvira Massaro, Barbara Galano, Valentina Ricucci, Bianca Bruzzone, Alexander Domnich

**Affiliations:** 1 Department of Health Sciences, University of Genoa, 16132 Genoa, Italy; 2799117@studenti.unige.it (M.S.); 4789907@studenti.unige.it (G.G.); elvira.massaro@edu.unige.it (E.M.); 2 Hygiene Unit, San Martino Policlinico Hospital, IRCCS for Oncology and Neurosciences, 16132 Genoa, Italy; miriana.lucente@hsanmartino.it (M.L.); chiara.fraccalvieri@hsanmartino.it (C.F.); nadia.randazzo@hsanmartino.it (N.R.); barbara.galano@hsanmartino.it (B.G.); valentina.ricucci@hsanmartino.it (V.R.); bianca.bruzzone@hsanmartino.it (B.B.)

**Keywords:** dengue, arboviruses, diagnosis, molecular diagnostics, RT2-PCR, multiplex RT2-PCR, diagnostic accuracy

## Abstract

*Background and Objectives*: The steady spread of dengue virus (DENV) poses a profound public health threat worldwide. Reverse transcription real-time polymerase chain reaction (RT2-PCR) has been increasingly recognized as a reference method for the diagnosis of acute dengue infection. The goal of this study was to assess the diagnostic accuracy of five different RT2-PCR kits for the detection of DENV in a historically processed set of sera samples. *Materials and Methods*: In this retrospective study, 25 sera samples from routinely processed unique adult patients with a known DENV status (previously tested in both molecular and serological assays) were tested in parallel using four conventional (RealStar Dengue PCR Kit 3.0, Clonit’ngo Zika, Dengue & Chikungunya, BioPerfectus Zika Virus/Dengue Virus/Chikungunya Virus Real Time PCR Kit and Novaplex Tropical fever virus) and one sample-to-result (STANDARD M10 Arbovirus Panel) RT2-PCR assays. Additionally, an end-point dilution analysis was conducted in quintuplicate on six serial dilutions of an RNA preparation obtained from a culture-grown DENV serotype 1 strain for a total of 150 tests. *Results*: The overall accuracy of the evaluated tests ranged from 84% to 100%. In particular, the sensitivity of three conventional RT2-PCR assays (RealStar, Clonit’ngo and Novaplex) was 100% (95% CI: 79.6–100%), while it was lower (73.3%; 95% CI: 48.1–89.1%) for the BioPerfectus kit. The sample-to-result STANDARD M10 panel performed comparatively well, showing a sensitivity of 92.9% (95% CI: 68.5–98.7%). No false positive results were registered in any assay. The end-point dilution analysis suggested that the RealStar kit had the lowest limit of detection. *Conclusions*: Available RT2-PCR kits for the detection of DENV are highly specific and generally sensitive and, therefore, their implementation in diagnostic pathways is advisable.

## 1. Introduction

Dengue virus (DENV), which belongs to the family *Flaviviridae*, is the most widespread mosquito-borne viral infection affecting humans. The virus is mainly transmitted through the bite of infected female *Aedes* mosquitoes, particularly *Aedes aegypti* and *Aedes albopictus* [1,2,3]. Four distinct serotypes of DENV are known (DENV-1, DENV-2, DENV-3, DENV-4), and it is possible to become infected several times with different serotypes [3,4]. Clinical manifestations of dengue infection vary significantly, ranging from asymptomatic to severe infection with multi-organ failure [5,6] and death in up to 13% of untreated patients [7]. However, there is no specific treatment for dengue [1]. Owing to the phenomenon of antibody-dependent enhancement, subsequent reinfection with a different serotype may expose one to the risk of developing a severe disease with a potentially fatal outcome [3,8].

Historically, DENV was considered endemic in several tropical and subtropical countries; however, ongoing climate change, rapid urbanization, globalization and increased international travel and lack of effective mosquito control measures are responsible for an increasing number of cases worldwide [9,10]. In particular, when environmental conditions are favorable and competent vectors are present, imported viruses can trigger local viral transmission [11]. According to the World Health Organization (WHO) [12], the global number of dengue cases is increasing: as of 30 April 2024, more than 7.6 million dengue cases have been reported in 2024, including 3.4 million confirmed cases and over 3000 deaths. The major increase has been seen in the WHO Regions of the Americas and South-East Asia [12]. Analogously, in several European countries, including Italy, an increasing number of autochthonous cases of DENV have been reported in recent years, not associated with travel, but probably due to the establishment of *Aedes albopictus* on the continent [2,11,13].

Similarly to many other countries, Italy has enhanced surveillance of dengue since 2012. The National Institute of Health issues regular updates on confirmed cases, described by type (autochthonous or imported), patient characteristics (e.g., sex, age) and lethal outcomes. In 2023, Italy reported 388 cases, of which 82 were autochthonous and 295 imported. In the first half of 2024, 259 cases were reported, all of which were imported [14].

Accurate laboratory diagnosis of dengue is essential for the clinical management of patients, surveillance purposes and effective control measures. Indeed, diagnostic accuracy of the proposed clinical criteria for dengue is poor [15]. A variety of direct (identification of the virus) and indirect (identification of virus-specific antibodies) virological methods for DENV have been developed [16,17,18]. The United States (US) Centers for Disease Control and Prevention (CDC) recommends [19] that for the acute (first 7 days after symptom onset) dengue diagnosis two strategies may be adopted: (i) rapid immunochromatographic antigen tests targeting non-structural glycoprotein 1 (NS1) plus detection of IgM through antibody capture enzyme-linked immunosorbent assay (ELISA) and (ii) nucleic acid amplification tests like reverse transcription real-time polymerase chain reaction (RT2-PCR) plus IgM serology [19]. However, each of these methods have important drawbacks. For instance, although ELISA IgM is often considered the preferred diagnostic method [20] and is invaluable for surveillance purposes [21], IgM is only detectable in a period starting from 4–5 days after symptom onset to the following approximately 12 weeks [19]. This means that IgM ELISA tests would often provide false negative results during the first most critical days of the disease and are, therefore, not useful for clinical decision making [21]. On the other hand, IgM is also not very specific and may determine false positive results caused by nonspecific reactivity [22]. NS1 antigen testing offers several advantages, such as low cost, ease of use and fast results, making this type of test efficient for endemic, high incidence and resource-limited areas [21,23,24]. On the other hand, sensitivity of the available NS1 antigen tests vary significantly (from 14.7% to 100% [20]), depends on the DENV serotype and may be lower for secondary infections [24]. In this regard, combining direct and indirect methods improves diagnostic accuracy and increases the diagnostic window [19,24].

Among other diagnostic tests, RT2-PCR for detecting DENV RNA is considered the most sensitive and specific method [25,26]. Although RT2-PCR can be generally used only at reference laboratories, as it usually requires specialized and trained personnel, expensive equipment and complex sample preparation procedures [25,26], it may have further advantages, such as DENV serotyping and availability of multi-viral panels for the differential diagnosis of different arboviral infections. Furthermore, RT2-PCR technology is in steady evolution, and an increasing number of manufacturers offer ready-to-use closed systems, which are very simple to work with and provide results in as little as one hour [27,28,29].

Considering both an important increase in dengue cases registered in the last five years [12] and recent license of effective vaccines [30], the demand for and availability of RT2-PCR assays will likely increase in the upcoming years. In a highly competitive market of molecular diagnostics, manufacturers search to differentiate their products by reducing turnaround times and improving efficiency and diagnostic accuracy. However, the initial validation of a novel kit performed by the manufacturer, typically on well-characterized samples, may not accurately reflect its diagnostic value. In this regard, independent external validation studies conducted in real clinical practice are vital. This study, which was carried out in the context of the internal optimization of the diagnostics of arboviruses, aimed to evaluate performance of five RT2-PCR kits for the detection of DENV in sera samples.

## 2. Materials and Methods

### 2.1. Study Design and Samples

To evaluate clinical diagnostic accuracy, a retrospective design was applied and, on the basis of the availability of samples and kits, a total of 25 deidentified sera samples with a known DENV status were identified and re-tested in parallel in each RT2-PCR assay evaluated. Of these latter samples, 15 historically processed and cryoconserved (at −80 °C) sera samples were positive for DENV, while the remaining 10 were negative. In particular, positive samples were collected from symptomatic adults admitted to the San Martino Research Hospital (Genoa, Italy) and tested positive in both ELISA IgM and standard-of-care RT2-PCR (earlier versions of the RealStar Dengue PCR Kit by Altona Diagnostics; Hamburg, Germany). These samples were also serotyped using the RealStar Dengue Type RT2-PCR Kit 1.0 (Altona Diagnostics; Hamburg, Germany) for research use only (RUO).

In the second part of the study, which was performed in order to establish relative analytical sensitivity, an end-point dilution analysis was conducted. For this purpose, six serial half-log (from 1:1 to 1:316) dilutions of a DENV-1 RNA preparation were tested in quintuplicate (i.e., five replicates for each dilution) in each assay. We defined the end-point dilution as the lowest dilution at which all five replicates tested positive [31]. DENV-1 RNA preparation was obtained from a DENV-1 strain grown in Vero cells, which were cultured in MEM 1X + GlutaMAX (+Earle’s salts + 25 mM HEPES) supplemented with stromal vascular fraction, antibiotics and amino acids. The virus supernatant was harvested when a prominent cytopathic effect was observed. The virus was doubly inactivated with both heat and chaotropic denaturants (guanidine hydrochloride in Buffer AL by Qiagen; Hilden, Germany) and diluted with plasma. DENV-1 RNA preparations were made by the Italian Institute of Health within a national quality control program for DENV diagnostics.

### 2.2. RT2-PCR Assays Evaluated

Five different RT2-PCR kits were assessed in this study, namely the RealStar Dengue PCR Kit 3.0 (Altona Diagnostics; Hamburg, Germany), Clonit’ngo Zika, Dengue & Chikungunya (Clonit; Milan, Italy), BioPerfectus Zika Virus/Dengue Virus/Chikungunya Virus Real Time PCR Kit (BioPerfectus Technologies; Taizhou, China), Novaplex Tropical fever virus (Seegene; Seoul, Republic of Korea) and the STANDARD M10 Arbovirus Panel (SD Biosensor; Seoul, Republic of Korea). Henceforth, these assays will be referred to as RealStar, Clonit’ngo, BioPerfectus, Novaplex and M10, respectively.

The assays evaluated differ in several characteristics, such as ease of use, number of analytes and time-to-result. For instance, while the RealStar kit detects only DENV, while the other four kits target also Zika (Clonit’ngo, BioPerfectus, Novaplex and M10), Chikungunya (Clonit’ngo, BioPerfectus, Novaplex and M10), West Nile (Novaplex and M10) and Yellow Fever (M10) viruses. Differently from other kits, the M10 assay is performed on a fully automated STANDARD M10 point-of-care platform, being an all-in-one cartridge-based RT2-PCR that integrates all steps in a closed system. M10 is also the only kit that differentiates between the four DENV serotypes. The other four RT2-PCR assays are more conventional, as their operational workflow includes separately performed RNA extraction and amplification steps. Novaplex was the only RUO kit.

Testing was performed according to the manufacturer’s instructions. Briefly, for the four conventional RT2-PCR kits (RealStar, Clonit’ngo, BioPerfectus and Novaplex), RNA extraction was performed on the automated ELITe InGenius workstation using the ready-to-use cartridge ELITe InGenius SP 200 (ELITechGroup; Puteaux, France). For this step, a sample volume of 200 µL was eluted in 100 µL of elution buffer. The time for extraction was approximately 30 min. RNA viral amplification was then performed on the CFX96 instrument (Bio-Rad Laboratories; Hercules, CA, USA) according to the thermic profile suggested by the manufacturer of each kit. The run-on times were 135, 102, 85 and 115 min for the RealStar, Clonit’ngo, BioPerfectus and Novaplex assays, respectively.

As noted earlier, the M10 assay is a fully automated RT2-PCR kit, which requires the addition of 600 µL of sample into the all-in-one cartridge, and results are available in 60 min.

A more detailed description of the amplification protocols and thermic profiles of single kits is reported in Supplementary Materials, Table S1.

### 2.3. Data Analysis

Relative diagnostic accuracy parameters included the overall accuracy, sensitivity and specificity with 95% confidence intervals (CIs). A generalized linear mixed model was applied to investigate the association between the assay and cycle threshold (Ct) values. Assay-specific Ct means were separated with post-hoc Tukey contrasts. All analyses were performed in R stats packages v. 4.1.0 (R Core Team, Vienna, Austria) and Excel v. 2408 (Microsoft, Redmond, WA, USA).

## 3. Results

Twenty-five clinical samples were tested in five RT2-PCR assays, of which fifteen (60%) were known to be positive. Of these latter samples, most (47%; 7/15) belonged to DENV-1, while DENV-2 (13%; 2/15), DENV-3 (27%; 4/15) and DENV-4 (13%; 2/15) were less frequent. One positive sample could be not tested with the M10 kit, since the residual volume was insufficient (<600 µL). Therefore, the results of 124 tests were analyzed.

As shown in Table 1, all RT2-PCR kits showed perfect (100%; 95% CI: 72.3–100%) specificities with no false positive results. With regard to sensitivity, the Novaplex, Clonit’ngo and RealStar kits identified correctly all 15 positive samples with a corresponding sensitivity parameter of 100% (95% CI: 86.7–100%). The M10 assay produced one (1/14; 7.1%) false negative result associated with a DENV-2-positive specimen, and its sensitivity was estimated at 92.9% (95% CI: 68.5–98.7%). In terms of serotype attribution, the M10 assay correctly identified serotypes of the remaining 14 positive samples. Finally, the false negative rate was higher (4/15; 26.7%) for the BioPerfectus kit and its sensitivity was 73.3% (95% CI: 48.1–89.1%).

Table 2 reassumes data on discordant results that concern four samples belonging to DENV-2 (*n* = 2), DENV-1 (*n* = 1) and DENV-3 (*n* = 1). All of these samples had comparatively low viral loads (Ct ≥ 32). Notably, the BioPerfectus kit did not detect both DENV-2-positive samples.

**Figure 1:**
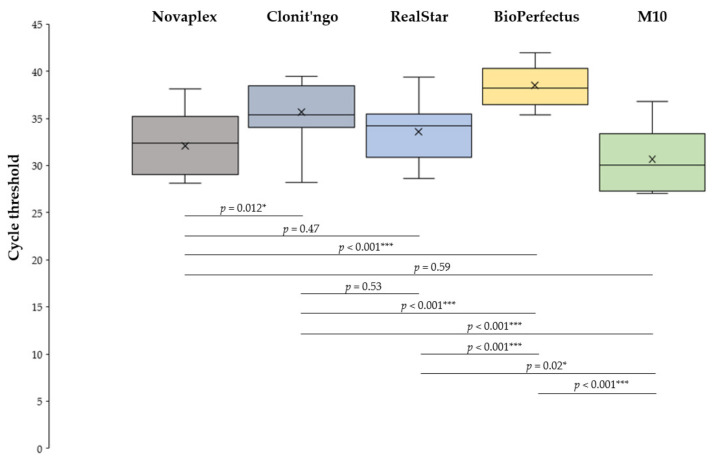
Distributions of cycle threshold (Ct) values, by assay. * *p* < 0.05; *** *p* < 0.001. All *p*-values are adjusted for multiple comparisons.

The distributions of Ct values provided by the five assays differed (*p* < 0.001), and several pairwise comparisons proved statistically significant (Figure 1). In particular, the lowest average Ct values were observed for the M10, Novaplex and RealStar assays, which were significantly lower (*P*_adj_ < 0.001) compared with the BioPerfectus kit.

In the analysis of serial dilutions (Table 3), RealStar (end-point dilution: 1:100) followed by Novaplex and M10 (end-point dilution: 1:31.6 for both) were deemed the most sensitive assays. Conversely, the BioPerfectus kit performed well only at the initial undiluted DENV concentration. Although the Clonit’ngo assay detected DENV-1 at 1:10 dilution in 80% (4/5) of replicates, its end-point dilution was 1:1 (Table 1).

## 4. Discussion

In this study, we evaluated five different RT2-PCR assays, most of which have been externally validated for the first time. Our principal findings may be summarized as follows: (i) commercially available RT2-PCR kits are generally accurate in detecting DENV in sera samples, though their analytical sensitivity varies; (ii) performance of multiplex RT2-PCR for the simultaneous detection of DENV and other arboviruses is similar to that of singleplex assays targeting DENV only; (iii) diagnostic accuracy of a rapid sample-to-answer kit is acceptable and may be even higher compared with some conventional laboratory-based assays.

RT2-PCR for the detection of DENV has been increasingly recognized as a reference method for the diagnosis of acute dengue, being both highly specific and sensitive [25,26]. Indeed, while historically DENV isolation in cell culture was considered the “gold standard”, cultural methods have been gradually replaced by the RT2-PCR due to the suboptimal sensitivity and relatively long turnaround times of the former [32]. Results of our study corroborate this trend: compared with a composite reference standard (i.e., discharge diagnosis plus previously documented positivity), four of five RT2-PCR kits showed a sensitivity ≥92% (while three kits showed perfect sensitivity), and all assays were perfectly specific.

Currently, a few studies [33,34,35,36,37] have validated commercially available RT2-PCR assays for the detection of DENV, and most of these used different versions of the RealStar kit. For instance, a Malaysian study [33] found that sensitivity of both the RealStar and GenoAmp Trioplex Real-Time RT2-PCR Zika/Den/Chiku (Mediven; Palau Pinang, Malaysia) kits was 90.3% (28/31), while it was somewhat lower (83.9%; 26/31) for the GenoAmp Real-Time RT2-PCR Dengue (Mediven; Palau Pinang, Malaysia) kit, which allows for DENV serotyping. All three kits showed perfect (100%; 5/5) specificity. Najioullah and colleagues [34] quantified the sensitivity of four different RT2-PCR kits, highlighting notable differences in their performance. In particular, the estimated sensitivities for the RealStar, Geno-Sen’s dengue 1–4 real-time RT2-PCR (Genome Diagnostics; New Delhi, India) and the Simplexa dengue RT2-PCR assay (Focus Diagnostics; Cypress, CA, USA) were 83.3% (135/162), 85.2% (138/162) and 93.2% (151/162), respectively. In assessing specificity, there was one false positive result (1.4%; 1/70) associated with the RealStar assay. The fourth dengue virus general type real-time RT2-PCR Liferiver kit (Shanghai ZJ Bio-Tech Co; Shanghai, China) showed comparatively poor performance (sensitivity of 70.0%; 28/40) in the initial evaluation and was not tested further [34]. Kann et al. [35] compared diagnostic accuracy of an in-house RT2-PCR and two commercial kits, namely the RealStar and Tropical Fever Core multiplex Real-time PCR (Fast Track Diagnostics; Luxembourg) kits. The RealStar performed well in detecting all DENV serotypes: the sensitivity parameters for DENV-1, DENV-2, DENV-3 and DENV-4 were 86.7% (13/15), 86.2% (25/29), 100% (29/29) and 100% (3/3), respectively. The specificity was high (97.9–100%) for all serotypes. Conversely, the second kit performed well only in detecting DENV-1 (100%; 1/1) and DENV-3 (100%; 24/24), while its sensitivity for DENV-2 was null (0%; 0/16) [35]. Notably, in our study, the BioPerfectus kit failed to identify both available DENV-2-positive samples. To summarize, with few exceptions, previous research has established high diagnostic accuracy of different commercially available RT2-PCR kits for DENV detection, which is consistent with our results. Some between-assay differences in sensitivity parameters are likely ascribable to different limits of detection and relative distributions of samples with different viral loads.

Next, we showed that compared with the RealStar kit, which in our study was the only singleplex assay, multiplex panels generally show comparable performance. Adding analytes to singleplex assays increases technical complexity and does not guarantee high diagnostic yields, and interferences between single targets are also possible [38]. We found that the analytical sensitivity of the singleplex RealStar assay was the highest, suggesting its comparatively low limit of detection. A similar finding was reported by Luciani et al. [37]. On the other hand, the potential advantages of the multiplex arboviral panels are significant. Indeed, the simultaneous detection of multiple pathogens or serotypes in a single test improves operational efficiency, enhances access to routine testing for viruses for which testing had been available only at reference laboratories, reduces turnaround times and overall costs [39,40]. Different arboviral infections share most signs and symptoms, which are often non-specific, and, in general, they are challenging to diagnose. Clinical differential diagnosis among single viral etiologies is typically not possible [41]. In several countries, such as Brazil, multiple arboviruses co-circulate and, even in narrow geographic areas the hotspots of DENV, Zika and Chikungunya viruses overlap [42,43]. Furthermore, co-infections between different DENV serotypes [44] and between DENV and other arboviruses [45] are not rare. Our results endorse a wider use of multiplex arboviral panels.

Among other kits evaluated, M10 was the only rapid sample-to-result all-in-one assay, which is performed using a miniaturized molecular diagnostics system. In fact, this platform was conceived for the point-of-care setting, and the M10 family of RT2-PCR kits has been extensively validated for SARS-CoV-2 and other respiratory viruses [46,47,48], mpox [49], multidrug-resistant tuberculosis [50] and *Clostridioides difficile* [51]. Compared with conventional RT2-PCRs, the use of this kit reduces the sample-to-answer time by 2–3 times, which may be very useful in some settings such as critical care. In our study, M10 proved highly specific (100%) and showed an acceptable sensitivity of 93%. Notably, the sensitivity of M10 was even higher compared with one conventional RT2-PCR kit. M10 is also among few multiplex panels developed not only to detect multiple arboviruses but also to serotype DENV; this latter feature is essential for virologic surveillance.

This study suffers from important limitations. Firstly, owing to a paucity of cases in the study area, a limited number of positive samples were available. This determined imprecise point estimates with relatively wide 95% CIs. Analogously, since the number of samples positive for single DENV serotypes was low, we decided against calculating serotype-specific accuracy parameters. Secondly, owing to a limited number of kits available, we were not able to test a higher number of negative samples and, therefore, the reported point estimates for specificity are likely inflated. For the same reason, we did not assess other validation parameters, such as analytical specificity and reproducibility. Thirdly, owing to the retrospective design, the calculation of predictive values was judged unfeasible. More generally, prospective studies of diagnostic test accuracy have several advantages over retrospective ones, especially if the prevalence of disease is low [52]. Larger prospective studies of the commercially available RT2-PCR assays for detecting DENV in fresh samples are warranted. Finally, the available set of DENV-positive samples may be representative of the study area and probably other countries with a comparatively low DENV incidence. Our results should be confirmed by studies conducted in geographic areas with high incidence.

In conclusion, this study showed that commercially available RT2-PCR kits for the detection of DENV generally have high diagnostic accuracy and, therefore, their wider adoption is advisable. In the context of a lean laboratory, multiplex arboviral RT2-PCR panels were found to be accurate and may optimize workplaces and workflows. However, it should be acknowledged that DENV RT2-PCR assays cost more than other techniques like antigen tests, and most of them require sophisticated laboratory equipment and qualified personnel; this may hamper the adoption of RT2-PCR in resource-constrained settings. Setting-specific, cost-consequence and budget impact analyses within the health technology assessment (HTA) framework may be helpful.

## Figures and Tables

**Table 1:** Accuracy, sensitivity and specificity of the RT2-PCR kits for the detection of dengue virus in clinical samples, by diagnostic accuracy parameter and assay.

Assay	Accuracy		Sensitivity		Specificity	
	% (*n*/*N*)	95% CI	% (*n*/*N*)	95% CI	% (*n*/*N*)	95% CI
RealStar	100 (25/25)	86.7–100	100 (15/15)	79.6–100	100 (10/10)	72.3–100
Novaplex	100 (25/25)	86.7–100	100 (15/15)	79.6–100	100 (10/10)	72.3–100
Clonit’ngo	100 (25/25)	86.7–100	100 (15/15)	79.6–100	100 (10/10)	72.3–100
BioPerfectus	84.0 (21/25)	65.4–93.6	73.3 (11/15)	48.1–89.1	100 (10/10)	72.3–100
M10	95.8 (23/24)	79.8–99.3	92.9 (13/14)	68.5–98.7	100 (10/10)	72.3–100

**Table 2:** Discordant results observed in the study of clinical samples for the detection of dengue virus.

Sample	Serotype	Qualitative Result (Cycle Threshold)				
		RealStar	Novaplex	Clonit’ngo	BioPerfectus	M10
A	1	+ (37.49)	+ (33.09)	+ (39.03)	Not detected	+ (32.02)
B	2	+ (36.42)	+ (35.31)	+ (38.10)	Not detected	+ (36.44)
C	2	+ (33.07)	+ (35.07)	+ (38.84)	Not detected	Not detected
D	3	+ (38.93)	+ (37.76)	+ (38.70)	Not detected	+ (33.45)

**Table 3:** Detection rates and cycle threshold values of a culture-grown serotype 1 dengue virus RNA preparation at different half-log dilutions, by assay.

Dilution	Parameter	% (*n*/*N*)				
		RealStar	Novaplex	Clonit’ngo	BioPerfectus	M10
1:1	Detected/Total	100 (5/5)	100 (5/5)	100 (5/5)	100 (5/5)	100 (5/5)
	Mean Ct (range)	32.26 (30.65–33.53)	31.29 (30.69–31.92)	36.22 (35.86–36.75)	37.06 (35.38–38.50)	29.73 (29.26–30.60)
1:3.16	Detected/Total	100 (5/5)	100 (5/5)	80 (4/5)	40 (2/5)	100 (5/5)
	Mean Ct (range)	33.95 (32.09–35.33)	32.89 (32.53–33.60)	38.28 (36.84–39.78)	39.81 (39.31–40.30)	31.52 (31.17–31.95)
1:10	Detected/Total	100 (5/5)	100 (5/5)	80 (4/5)	40 (2/5)	100 (5/5)
	Mean Ct (range)	35.54 (34.24–36.43)	35.02 (34.30–36.30)	38.34 (38.01–39.07)	39.59 (37.49–41.61)	33.04 (31.92–33.95)
1:31.6	Detected/Total	100 (5/5)	100 (5/5)	40 (2/5)	20 (1/5)	100 (5/5)
	Mean Ct (range)	37.30 (36.42–38.04)	37.03 (35.80–38.51)	39.23 (39.17–39.29)	40.88 (NA)	34.61 (33.28–35.93)
1:100	Detected/Total	100 (5/5)	80 (4/5)	0 (0/5)	0 (0/5)	60 (3/5)
	Mean Ct (range)	37.57 (36.93–38.71)	38.09 (36.67–39.31)	NA	NA	35.51 (35.40–35.66)
1:316	Detected/Total	0 (0/5)	0 (0/5)	0 (0/5)	0 (0/5)	0 (0/5)
	Mean Ct (range)	NA	NA	NA	NA	NA

Ct, cycle threshold; NA, not available.

## Data Availability

All relevant data are within the manuscript and associated Supplementary Materials. Further inquiries can be directed to the corresponding author.

## Supplementary Materials

The following supporting information can be downloaded at: https://www.mdpi.com/article/10.3390/medicina60091557/s1, Table S1: Synthesis of the amplification protocols used for the molecular assays evaluated.
