# Supplementary material for: Diagnostic Accuracy of Five Molecular Assays for the Detection of Dengue Virus
Source: Medicina (Kaunas). 2024 Sep 23;60(9):1557. doi: 10.3390/medicina60091557 (PMC11434457; doi:10.3390/medicina60091557)
Supplement: Supplementary file 1 [file medicina-60-01557-s001.zip › medicina-3196930-supplementary.pdf]

Table S1. Synthesis of the amplification protocols used for the molecular assays evaluated.

Assay	Protocol
RealStar Dengue PCR Kit 3.0 (Altona Diagnostics; Hamburg, Germany)	The kit provides with all the necessary reagents for preparing the reaction mix: 5 μl of Master A and 15 μl of Master B for a total volume of 20 μl for each sample. RNA volume required is 10 μl. The kit includes an internal control (IC), which is added during the extraction phase in proportion to 10% of the elution volume. Cycling conditions were: 55 °C for 20′ (RT step), 95 °C for 2′, 45 cycles at 95 °C for 15′′, 55 °C for 45′′ and 72°C for 15′′ (running time 135 min). Dengue detection was conducted at 55 °C on the FAM fluorophore.
Clonit'ngo Zika, Dengue & Chikungunya (Clonit; Milan, Italy)	The test is performed on strips containing the lyophile reaction mix reconstituted by adding 5 μl of Buffer B to each well. RNA volume required is 5 μl for a total volume of 20 μl. The kit does not include the addition of the IC during the extraction step, as it is already present in the lyophilic mix. Cycling conditions were: 45° C for 15′ (RT step), 95° C for 2′, 45 cycles at 95° C for 10′′, 60 °C for 50′′ (running time 102 min). The detection was conducted at 60 °C on the FAM fluorophore for Dengue, ROX for Chikungunya e Cy5 for Zika viruses.
Zika Virus/Dengue Virus/Chikungunya Virus Real Time PCR Kit (BioPerfectus Technologies; Jiangsu, China)	The reaction mixes (for a total of 20 μl) per sample are composed as follows: 7.5 μl of the PCR Buffer, 5 μl of the PCR Enzyme mix, and 7.5 μl of the Reaction mix. RNA volume required is 5 μl. The Human RNase P gene was used as IC. Cycling conditions were: 50 °C per 10′ (RT step), 95 °C per 5′, 45 cycles at 95 °C for 10′′, 60°C for 30′′ (running time 85 min). The detection was conducted at 60 °C on FAM for Dengue, VIC for Zika, and ROX for Chikungunya viruses.
Novaplex Tropical fever virus (Seegene; Seoul, Republic of Korea)	This kit is for research use only (RUO). The kit provides with an IC added during the sample extraction phase in proportion to 10% of the elution volume. The master mix contains 5 μl of MOM (amplification and detection reagent), 5 μl of Buffer and 5 μl of Enzyme. RNA volume required is 5 μl. Cycling conditions were: 50 °C for 20′ (RT step), 95 °C for 15′, 45 cycles to 95 °C for 10′′, 60 °C for 15′′ and 72 °C for 10′′ (running time 115 min). The detection was conducted at 60 °C and 72 °C on FAM for Dengue, HEX for Chikungunya, Cal Red 610 for Zika and Quasar 705 for West Nile viruses.
STANDARD M10 Arbovirus Panel (SD Biosensor; Seoul, Republic of Korea)	This kit is an all-in-one RT2-PCR that integrates all steps in a closed system and thus contains all primers, probes, internal control (IC) and other reagents in a single cartridge. The assay must be

	performed on a fully automated STANDARD M10 platform. Specific primers are labelled with the HEX probe to show positivity for DENV-1, DENV-2, DENV-4 and ZIKV, while the FAM probe shows positivity for DENV-3, Yellow Fever and West Nile viruses. The IC is labelled by Cy5 probe. The time-to-result is 60 min.

DENV, Dengue virus; IC, internal control.
